# Early androgen activity after birth determines the hypothalamic expression of androgen and estrogen receptors in adulthood in female but not in male rats

**DOI:** 10.1186/s12958-025-01430-y

**Published:** 2025-07-07

**Authors:** Rocío García-Úbeda, Jose Manuel Fernandez-Garcia, Ulises Primo, Daniela Grassi, Antonio Ballesta, Maria Angeles Arevalo, Paloma Collado, Helena Pinos, Beatriz Carrillo

**Affiliations:** 1 Department of Psychobiology, National University of Distance Education (UNED), C/ Juan del Rosal, 10. 28040, Madrid, Spain; 2 University Institute of Research-UNED-Institute of Health Carlos III (IMIENS), Madrid, Spain; 3 Faculty of Psychology, Villanueva University of Madrid, Madrid, Spain; 4 Department of Anatomy, Histology and Neuroscience, Autonoma University of Madrid, Madrid, Spain; 5 Biomedical Research Center, CIBER of Frailty and Healthy Aging (CIBERFES), Institute of Health Carlos III, Madrid, Spain; 6 Department of Psychobiology, Centro de Enseñanza Superior Cardenal Cisneros, Madrid, Spain; 7 Neuroactive Steroids Lab, Cajal Institute, CSIC, Madrid, Spain

**Keywords:** Androgen receptor, Aromatase, 5α-reductase, Gonadal steroids, Development, Sex differences, Rat

## Abstract

Gonadal steroids are involved in the organization and programming of several neural systems. The main objective of this study was to determine whether androgen activity in the early postnatal stage influenced the long-term expression of androgen and estrogen receptors in the hypothalamus. Androgen receptors (AR) and the main metabolic pathways of testosterone were inhibited using Flutamide, an AR inhibitor, Letrozole, an aromatase inhibitor, or Finasteride, a 5-alpha-reductase inhibitor, during the first five days of life in male and female Wistar rats. Hypothalamic hormonal receptors AR, and estradiol receptors (ER)α, and ERβ were analyzed by qPCR, and circulating hormone levels (testosterone, DHT, and estradiol) were measured using ELISA assay at P90. The inhibition of AR, 5α-reductase or aromatase did not alter the hypothalamic levels of hormone receptors in males. However, in females, blocking the androgen receptor increased the ERβ, while the inhibition of 5α-reductase decreased the ERα and the inhibition of aromatase increased AR and ERβ hypothalamic mRNA levels. Moreover, testosterone plasma levels decreased significantly in females independent of whether the AR, 5α-reductase, or aromatase were inhibited. However, only the inhibition of aromatase decreased circulating testosterone levels in males. Furthermore, higher plasma testosterone and DHT levels were detected in males compared to females. Our results highlight the influence of androgen activity during the first days of life in females on the long-term expression of androgen and estrogen receptors in the hypothalamus, which reaffirms the importance of studying both sexes to accurately explain the processes that determine the programming of neural systems during development.

## Highlights

Early postnatal androgenic activity programs steroid receptors in females.

Male sex steroid receptors do not depend on early postnatal androgenic activity.

Testosterone levels in adulthood depend on postnatal androgenic activity in females.

Androgenic activity differentially alters sex steroid receptors in the hypothalamus.

## Introduction

Numerous studies have confirmed the importance of the early stages of life for the proper development of the neural systems that control individual behaviour [1–3]. Among the factors involved in the differentiation and organization of the nervous system, the activity of the gonadal steroids during the pre and early postnatal stages has been shown to be relevant in determining the morphology and function of different brain networks, including reproductive and feeding systems [4–7].

The role of estrogens and androgens in the sexual differentiation of reproductive behaviors, and the morphology of the brain structures that support them, is well established [4, 5, 8–11]. Likewise, the participation of gonadal steroids in the programming of the feeding system has also been studied. Estrogen and androgen activity during the first days of life results in the differential development of several peptides involved in feeding systems in the brain in male and female rodents [6, 7, 12–15]. This data demonstrates that androgens and estrogens participate in the programming of cerebral networks differentially in males and females.

During the first days of life in rats, there is a considerable increase in the levels of testosterone, as well as an increase in the enzymes that metabolize it and the receptors on which it acts. Higher levels of testosterone have been observed in male rats at the end of the gestation period and on the first day of life [2]. However, it is important to note that in female rats, testosterone and DHT levels are also detectable in both plasma and brain tissues, including the hypothalamus, during the first days of life. Furthermore, it has been shown that the levels of these gonadal steroids in females are comparable to those in males during the first five days of life [16, 17]. In the hypothalamus, estrogen receptors can be detected around day 21 of gestation and their expression increases considerably in the first days of postnatal life [18] while androgen receptors are present in the hypothalamus and in other brain regions seven days before birth, progressively increasing from then on until they reach adult levels a few weeks after birth [19]. Moreover, the levels of the enzyme aromatase increase in the last days of gestation and the first days after birth [20–22]. As many authors have already pointed out, the rapid increase in estrogen and androgen activity that occurs at the end of gestation and during the first days of life is critical for the differentiation and programming of various neural networks [5, 22, 23]. In this regard, it is important to investigate the pathways through which gonadal steroids act in the brain.

During this programming, androgens and estrogens act through their own receptors, but while estradiol acts through the classical estradiol receptors (ER) α and ERβ, binding to the specific response elements (ERE), or through membrane receptors activating second messengers [24, 25] testosterone can directly activate the androgen receptor (AR) but can also indirectly activate the ERα or ERβ via its metabolites. On the one hand, testosterone can be metabolized either to estradiol by the enzyme P450-aromatase or to dihydrotestosterone (DHT) by the enzyme 5-α reductase. Similarly, like testosterone, DHT acts on the AR, but also through ERβ via one of its metabolites, 5α-androstane, 3β,17β-diol (3β-diol) [26]. Therefore, all these actions must be considered when studying the effects of gonadal steroids on the programming of brain circuits, since androgenic activity can act on both androgen and estrogen receptors and estrogenic activity depends on both estrogen and androgen aromatization.

The influence of gonadal hormones on the expression of estrogen and androgen receptors in the brain has previously been reported. Sustained exposure to estradiol decreased the expression of ERs in several brain regions, whereas DHT increased the expression of ERs in hypothalamic nuclei such as the arcuate nucleus (Arc) or the ventromedial hypothalamus (VMH) of adult female rats [27]. Moreover, AR and AR mRNA levels increase in several hypothalamic nuclei in male rats treated with estradiol [28, 29] while DHT or androgenic anabolic steroids upregulate AR expression in the VMH [30]. Similar effects were reported when estradiol treatment was administered to rats during the first five days of life, since AR protein and mRNA levels decreased in the hypothalamus of male rats in adulthood [31].

All these results show that androgenic and estrogenic activity affect the expression of gonadal receptors in different brain regions. Therefore, due to the importance of these hormones in the programming of different brain systems during the first days of life, it is important to study how the activity of these hormones influences the development of different brain parameters, specifically, the expression of the receptors through which these hormones act.

The effects of neonatal androgens in males are already well established. However, because androgens act through various mechanisms via their metabolites, the specific pathways, particularly in females, remain unclear. Therefore, the aim of this study was to clarify the mechanisms by which neonatal androgenic activity regulates the expression levels of gonadal steroid receptors in adulthood in both male and female rats. For this purpose, androgenic activity was severely reduced by inhibiting one of the following during the first five days of postnatal life; the AR, the P450-aromatase or 5-α reductase activity. Then, the AR, ERα or ERβ mRNA levels were analyzed, as well as the plasma levels of estradiol, testosterone and DHT, on postnatal day (P) 90. Due to the presence of androgenic activity during the first days of life in both males and females [16, 17, 22, 32]– [33], the present study was conducted in both sexes to determine whether this activity had differential long-term effects on the expression of gonadal steroid receptors in both sexes.

## Materials and methods

### Animals

Wistar rats were maintained in stable conditions of temperature, humidity and light (22 ± 2ºC; 55 ± 10% humidity; 12 h light/12 h darkness cycle, with light from 8:00 till 20:00), with food and water *ad libitum*. Throughout the study, animal care and handling practices were approved by our Institutional Bioethics Committee (UNED, Madrid), following the “Guidelines for the Use of Animals in Neuroscience Research”, developed by the Neuroscience Society, the European Union legislation (Council Directives 86/609/EEC and 2012/63/UE) and the Spanish Government legislation (RD 1201/2005). Special care was taken to keep the number of animals to a minimum and to minimize animal suffering.

For mating, a male was placed in a cage with two females for five days. The pregnant females were placed in individual plastic maternity cages with wood shavings as nesting material.

**Fig. 1:**
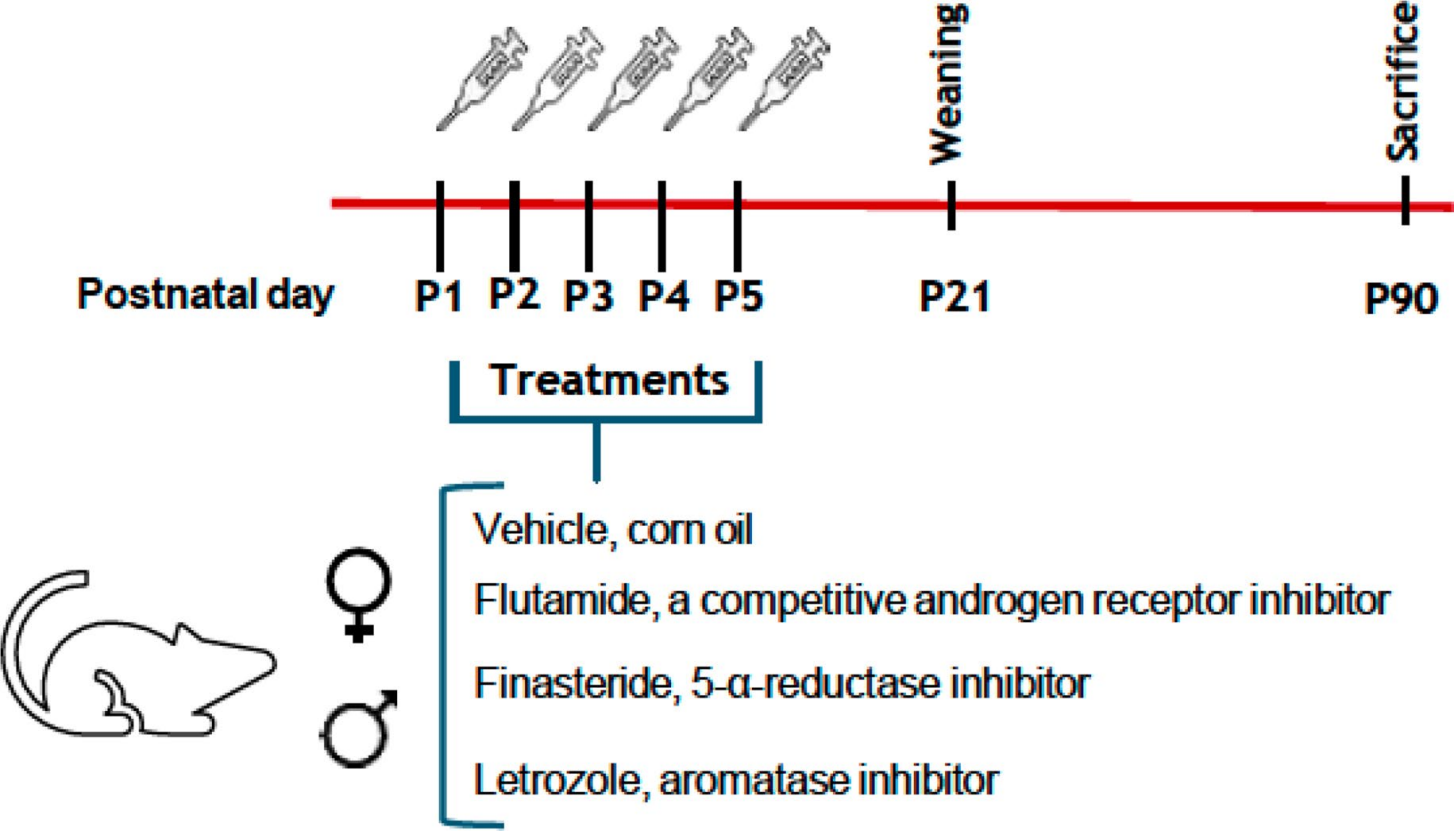
Schematic diagram of the experimental setup: male and female pups were treated during the first five postnatal days with flutamide, a competitive androgen receptor inhibitor, or finasteride, a 5α-reductase inhibitor, or letrozole, an aromatase inhibitor. Animals were sacrificed on postnatal day 90

On postnatal day 1 (P1), pups born on the same day were weighed, sexed, and randomly distributed (five males and five females per litter) among the nursing mothers and from P1 until P5, the pups were injected subcutaneously with a volume of 0.01 ml/kg of one of: vehicle (VH, corn oil), or flutamide, a competitive AR inhibitor (25 mg/kg; F9397, Merk), or finasteride, a 5-α-reductase (enzyme) inhibitor (5 mg/kg; NB-48–0403–100MG, Quimigen), or letrozole, the aromatase inhibitor (1 mg/kg; L6545, Merck). All doses were selected based on previous studies [34–38]. A diagram with the experimental setup and the molecular targets of the inhibitors is shown in Fig. 1. Consequently, the experimental groups were as follows: control males (CM = 5), control females (CF = 5), flutamide-treated males (FluM = 5), flutamide-treated females (FluF = 5), finasteride-treated males (FinM = 5), finasteride-treated females (FinF = 5), letrozole-treated males (LetM = 5) and letrozole-treated females (LetF = 5).

The extended description of the procedures used for this experiment is cited in the group’s previous articles [15]. Briefly, on weaning day (P21) all animals were housed individually. Only from the females, on P89 a vaginal smear was taken to determine the estrous phase. Those females in the diestrus phase on P90 were decapitated together with the same number of animals from the other groups. On P91 or P92, same procedure was followed to assure that all females in all groups were in the diestrus phase at the time of sacrifice. The animals were sacrificed by decapitation between 9:00 and 11:00 a.m. Trunk blood was collected in glass tubes containing ethylenediamine-tetra-acetic acid (EDTA), centrifuged for 15 min at 2000 g at 4 °C and the plasma was collected and stored in aliquots at − 80 °C. The hypothalamus was studied as a whole. After decapitating the animals, the brain was removed, and the hypothalamus was completely dissected. The hypothalamus appears in the ventral region of the brain as a well-differentiated, circular area, so its extraction can be performed with considerable precision. When the hypothalamus was removed, it was rapidly frozen in dry ice and stored at -80ºC until processing.

### RNA extraction and quantitative PCR (q-PCR)

Total RNA was extracted from the isolated tissues with Illustra RNAspin mini-RNA isolation kit (GE Healthcare) following the manufacturer’s instructions to measure AR, ERα, and ERβ mRNA, and Pgk1 as the housekeeping gene.

Total RNA concentration and purity were measured with a NanoDrop^®^ ND- 1000 UV-Vis Spectrophotometer (Thermo Scientific, Washington, DC, USA). A reverse transcription of 2 µg of RNA to cDNA was performed with the commercial kit for reverse transcription (RT) NZY TECH following the manufacturer’s recommendations, in a Tetrad 2 thermocycler (Peltier Thermal Cycler, Bio-Rad, California, USA) with the following conditions: 25ºC for 10 min, 37ºC for 50 min, 85ºC for 5 min, and 37ºC for 20 min with MMLV Reverse Transcriptase enzyme (Promega Biotech Iberia, Madrid, Spain) at a concentration of 200 µg/µL.

The q-PCR amplification was performed using the TaqMan^®^ Universal PCR Master Mix protocol (Applied Biosystems, Washington DC, USA) in the 7500 Real Time PCR System device (Applied Biosystems) and specific primers were used for each gene of interest (TaqMan Gene Expression Assay Mix kits). The conditions of the q-PCR were: 50º C for 2 min, 95º C for 10 min, and 40 cycles of amplification and extension (15 s at 95º C and 1 min at 60º C).

The primers (Applied Biosystems) used in the genetic expression study were: AR (Gg07164695_s1), ERα (Rn01640372_m1) and ERβ (Rn00562610_m1).

Each sample was amplified in duplicate. The expression values of each gene were normalized with the expression levels of the endogenous housekeeper Pgk1 (Rn00569117_m1), whose values did not vary between the experimental groups [39, 40]. The levels of relative genetic expression were analyzed using the ΔΔCT method and statistical analysis were performed on the resulting data.

### ELISAs

Plasma testosterone, DHT, and estradiol levels were measured in duplicate by ELISA following the manufacturer’s instructions. Absorbance in each well was measured with Tecan Infinite M2000 (Grödig, Austria). The ELISA kit CEA461Ge (Cloud-Clone Corp.) was used for the estradiol detection, with a detection range of 12.35–1000 pg/ml. Testosterone detection was performed with the ELISA kit CSB-E05100r (Cusabio), with a detection range of 0.13–25.6 ng/ml. The ELISA kit CEA443Ge (Cloud-Clone Corp, USA) was used for the DHT detection, range 30.9-2,500pg/ml.

### Analysis

Hypothalamic AR, ERα, and ERβ mRNA levels were analyzed as follows: to determine sexual dimorphism, ANOVAs between males and females with treatment as a factor were carried out. The significance level was set at *p* < 0.05. To determine intra-sex differences, male and female groups were analyzed independently using a one-way ANOVA, in which each treatment group was contrasted with its corresponding control group. The significance level was set at *p* < 0.05. The IBM Statistics Package for Social Sciences (SPSS) version 29 was used to perform the statistical analysis.

## Results

### AR, ERα and ERβ mRNA levels

#### Hypothalamic AR mRNA levels

The analysis of the hypothalamic AR mRNA levels showed no significant differences between CM and CF, indicating that there was no sexual dimorphism in this parameter. However, significant differences between FluM and FluF (F_(1,9)_: 6.801; *p* < 0.05) and LetM and LetF (F_(1,9)_: 85.874; *p* < 0.005) groups were detected, with females having higher hypothalamic AR mRNA levels than males (Table 1).

**Table 1:**
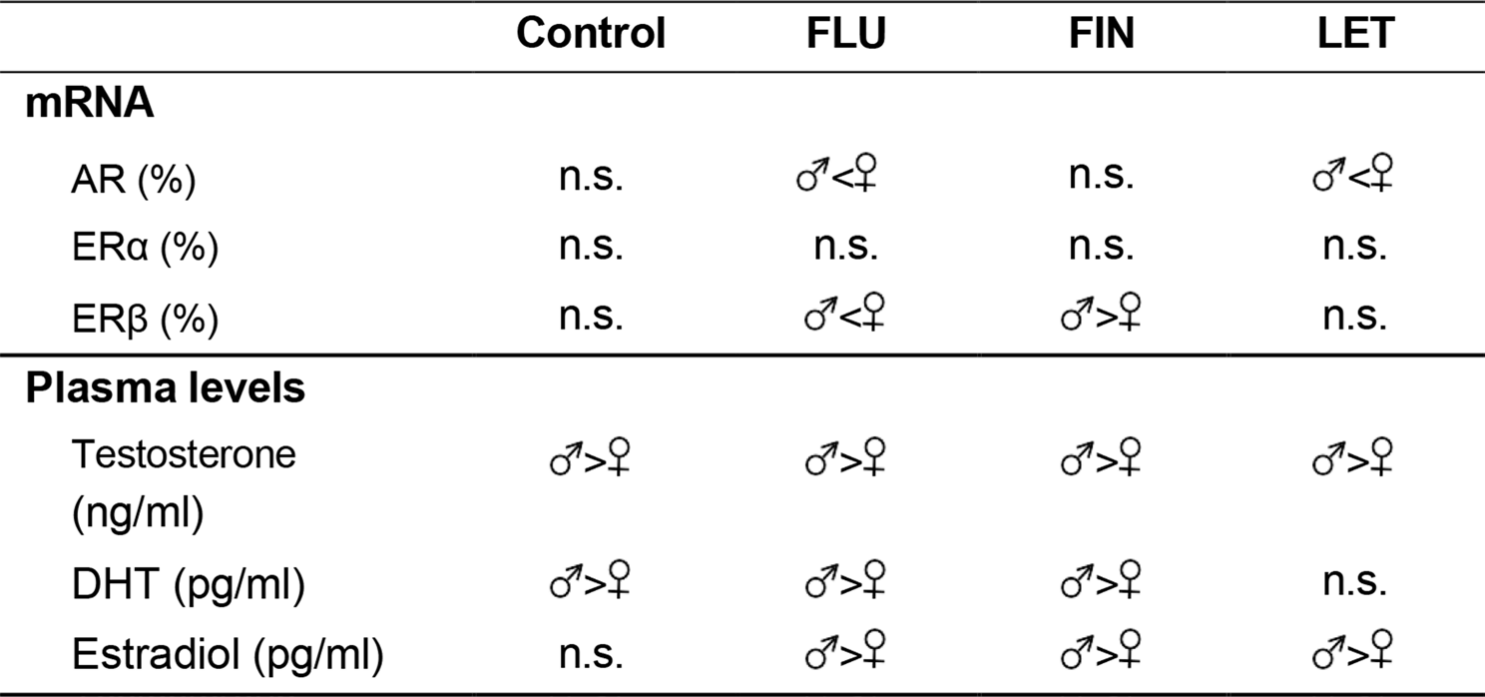
Summary of sex differences

**Fig. 2:**
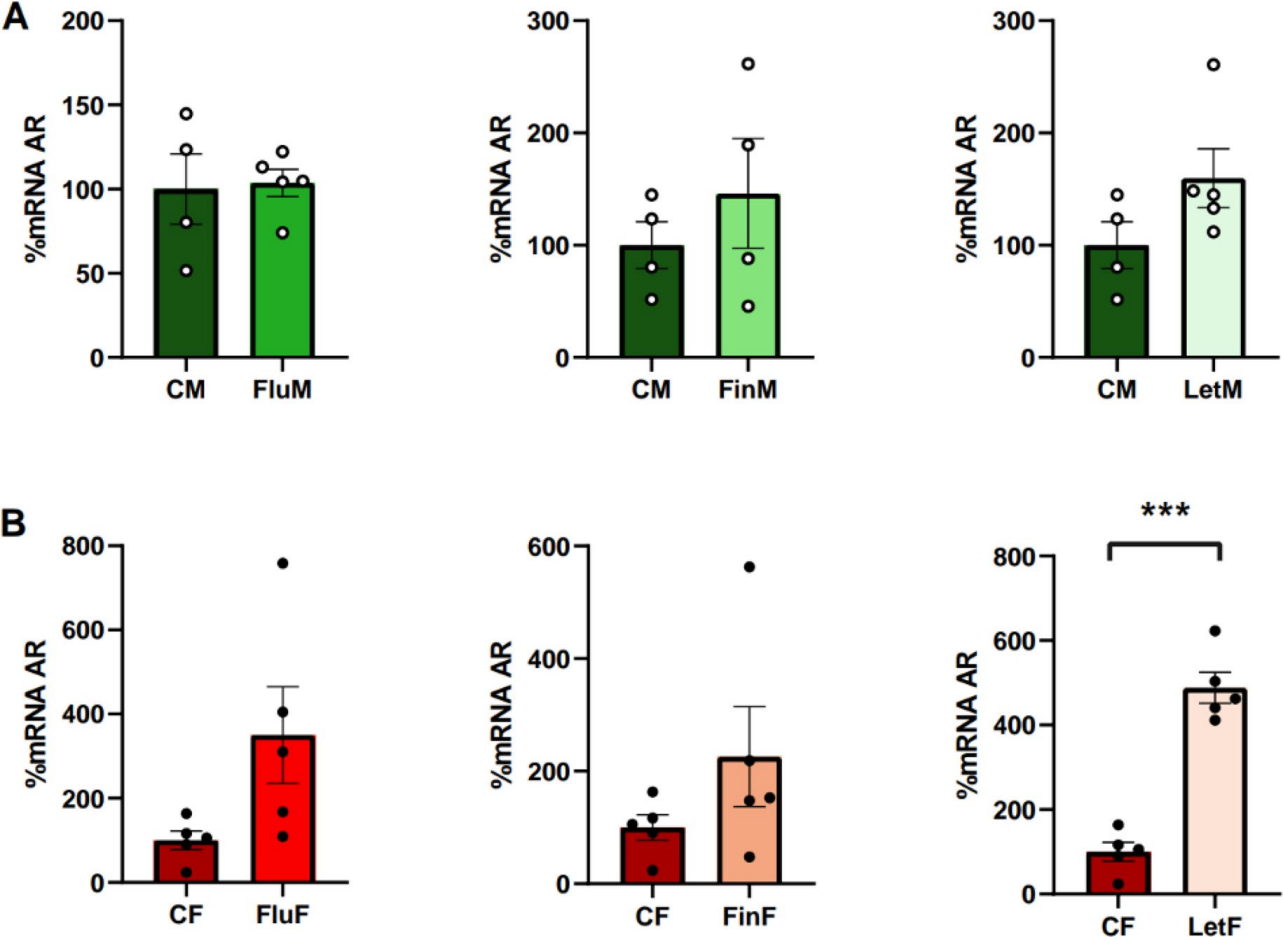
Bar charts of hypothalamic levels of AR, (**A**) when male groups, or (**B**) female groups were analyzed separately; *** show statistically significant differences of *p* < 0.001

When male groups were analyzed separately, no significant differences were detected (Fig. 2A) but in females, a significant increase in the hypothalamic AR mRNA levels was shown when aromatization was inhibited, since treatment with letrozole showed lower values in LetF compared with CF (F_(1,9)_: 80.582; *p* < 0.005) (Fig. 2B).

#### Hypothalamic ERα mRNA levels

Regarding sexual dimorphism, the analysis of the hypothalamic ERα mRNA levels showed no significant differences between males and females in either control or experimental groups (Table 1).

**Fig. 3:**
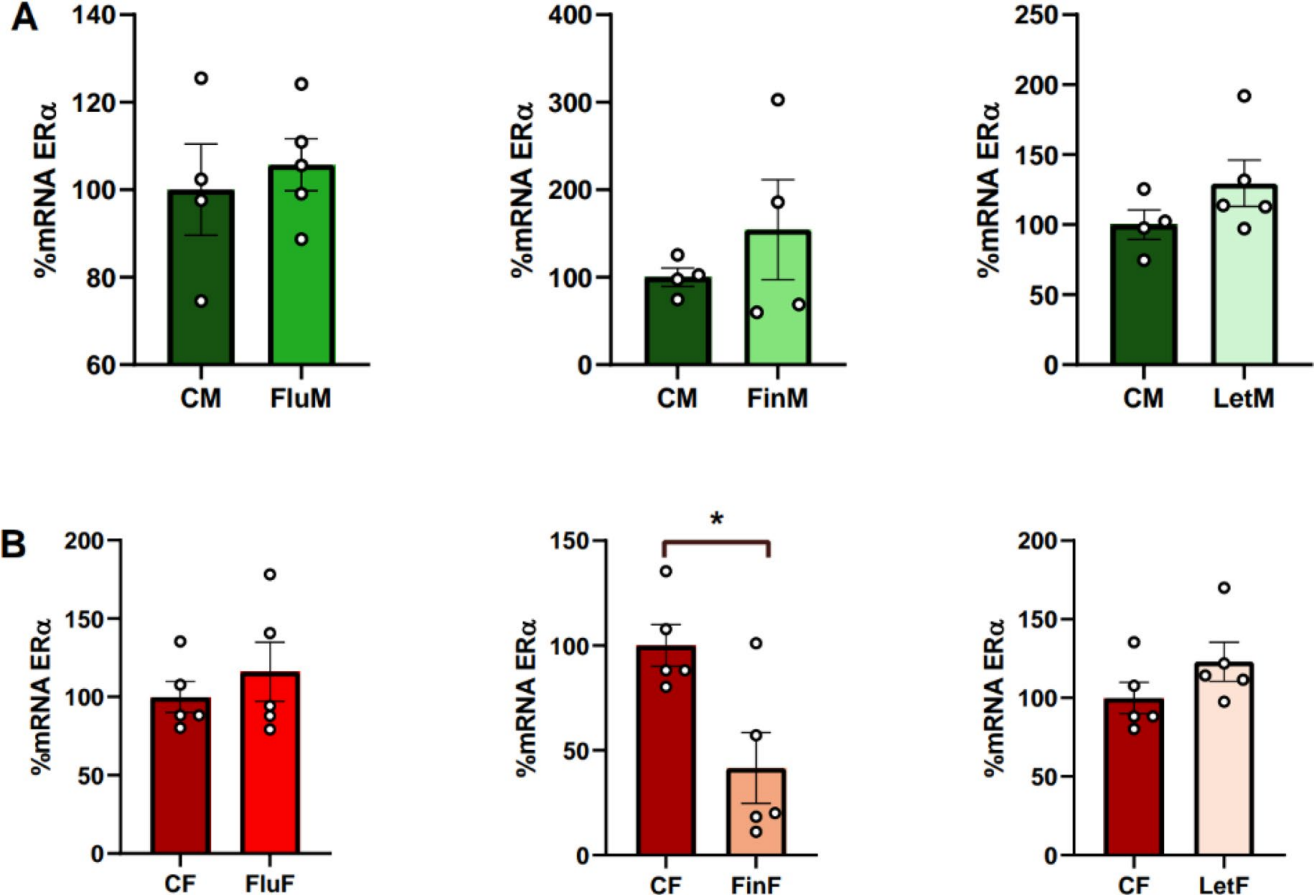
Bar charts of hypothalamic levels of ERα, **A**) when male groups, or **B**) female groups were analyzed separately; * show statistically significant differences of *p* < 0.05

When each sex was analyzed separately, no significant differences were found in male groups (Fig. 3A). However, when female groups were analyzed separately, significant differences were detected between CF and FinF (F_(1,9)_: 8.887; *p* < 0.05), with CF showing higher values of hypothalamic ERα hypothalamic levels than FinF (Fig. 3B).

#### Hypothalamic ERβ mRNA levels

The analysis of the hypothalamic ERβ mRNA levels showed no significant differences between CM and CF, indicating an absence of sexual dimorphism in this receptor. Nevertheless, significant differences were found between FluM and FluF (F_(1,9)_: 30.576; *p* < 0.005), with females showing higher values than males, and FinM and FinF (F_(1,8)_: 13.311; *p* < 0.01), with males showing higher values than females (Table 1).

**Fig. 4:**
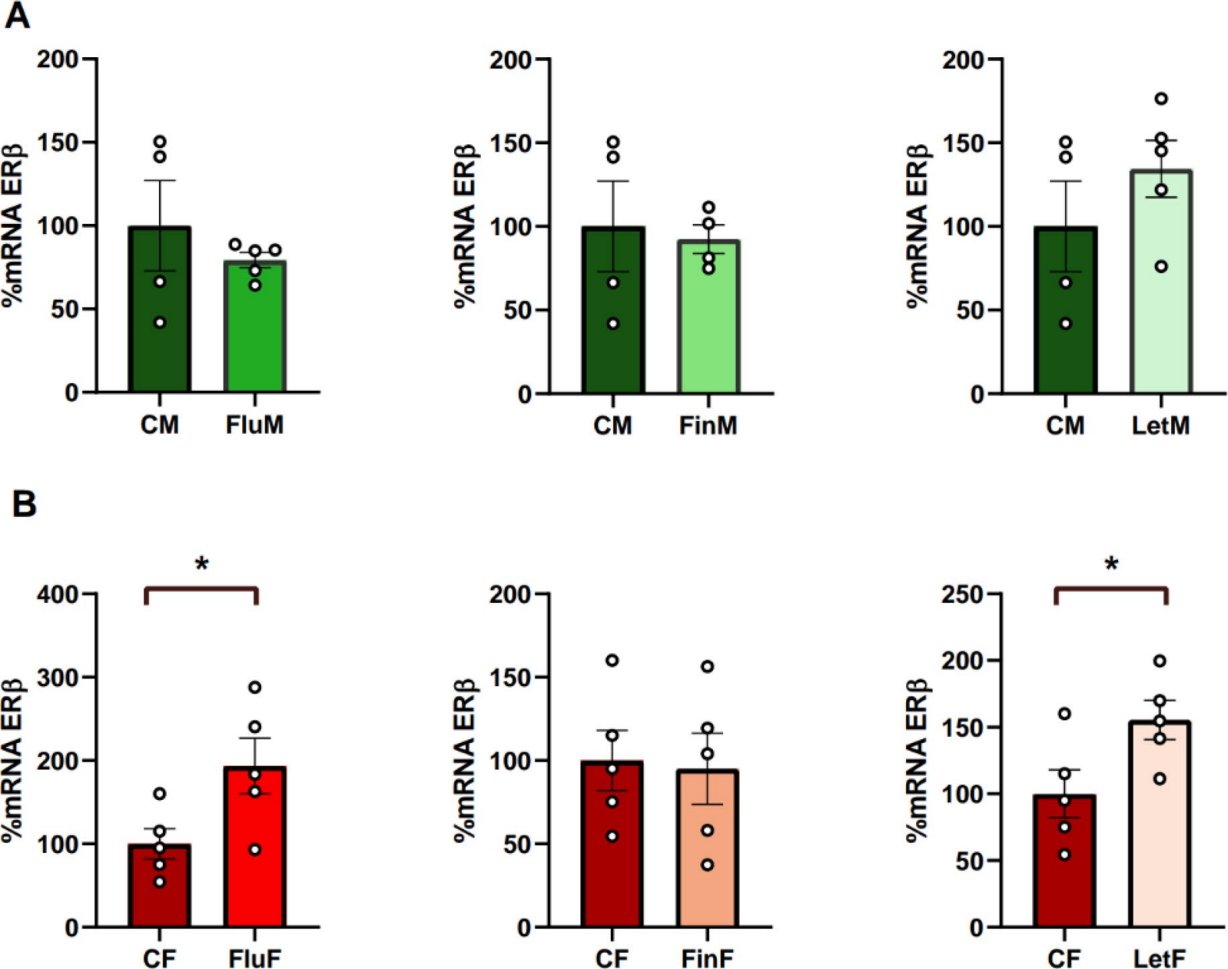
Bar charts of hypothalamic levels of ERβ, **A**) when male groups, or **B**) female groups were analyzed separately; * show statistically significant differences of *p* < 0.05

When each sex was analyzed separately, no significant differences were detected in the male groups (Fig. 4A) whereas in the female groups significant differences were detected between CF and FluF (F_(1,9)_: 6.099; *p* < 0.05) and CF and LetF (F_(1,9)_: 5.675; *p* < 0.05), with both experimental groups showing higher hypothalamic ERβ mRNA levels than the control group (Fig. 4B).

### Plasma hormone levels

#### Testosterone

Regarding sexual dimorphism, the analysis of the circulating levels of testosterone showed significant differences between the control groups, CM and CF (F_(1,8)_: 991.4; *p* < 0.005), as well as between all treatment groups: FluM and FluF (F_(1,9)_: 1026.144; *p* < 0.005), FinM and FinF (F_(1,9)_: 1722.378; *p* < 0.005) and LetM and LetF (F_(1,9)_: 501.589; *p* < 0.005), with male testosterone plasma levels being higher than those of females for all groups (Table 1).

**Fig. 5:**
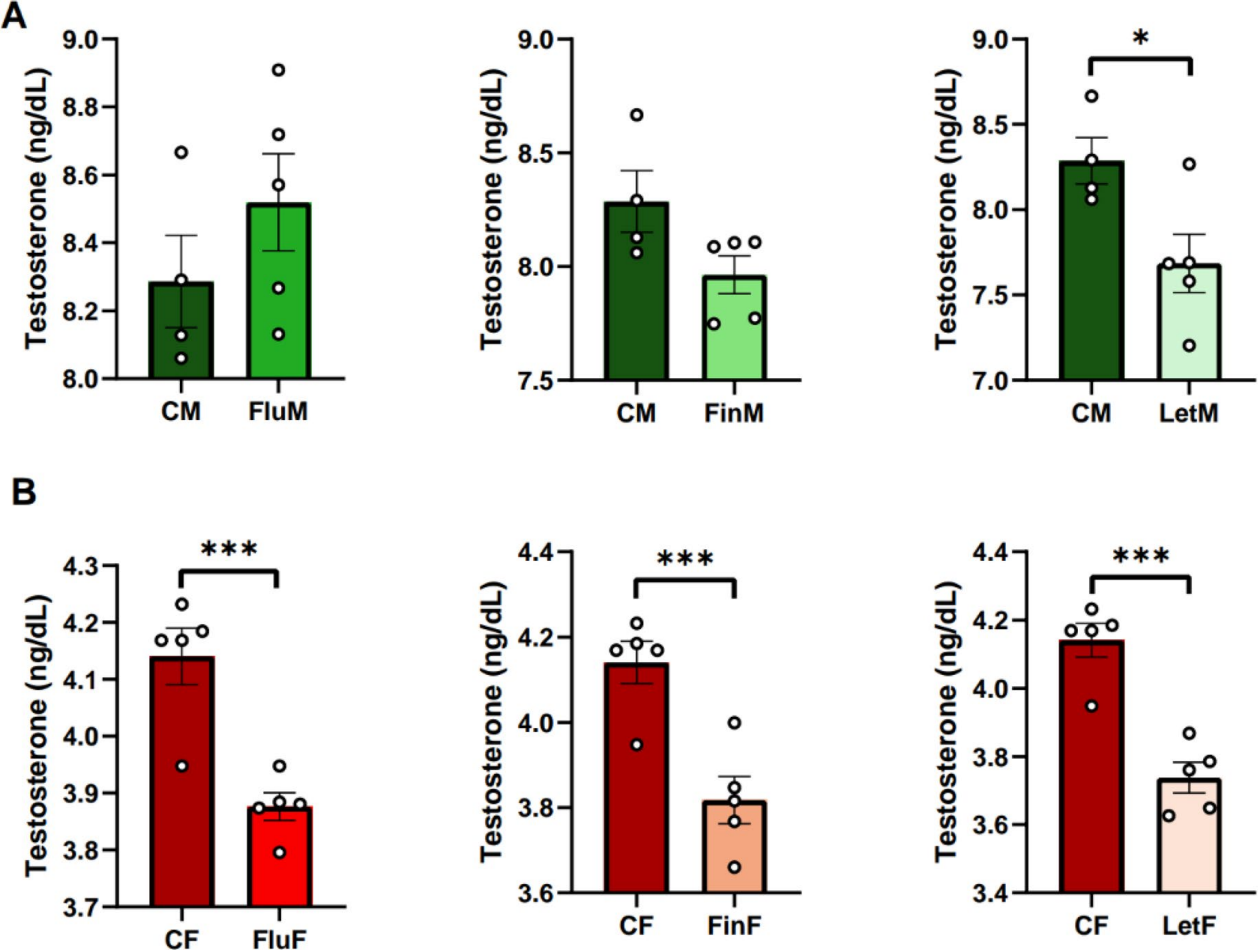
Bar charts of testosterone plasma levels, **A**) when male groups, or **B**) female groups were analyzed separately; * show statistically significant differences of *p* < 0.05; *** show statistically significant differences of *p* < 0.001

When each sex was analyzed separately, it was observed that males showed significant differences only between the CM and LetM groups (F_(1,8)_: 7.012; *p* < 0.05), with testosterone levels being higher in the control group than in LetM (Fig. 5A). However, all three treatments altered the circulating testosterone mRNA levels in females, since significant differences between CF and FluF (F_(1,9)_: 22.922; *p* < 0.005), CF and FinF (F_(1,9)_: 18.864; *p* < 0.005) and CF and LetF (F_(1,9)_: 36.295; *p* < 0.005) were observed, with circulating testosterone mRNA levels being consistently higher in the control group (Fig. 5B).

#### DHT

Regarding sexual dimorphism, the analysis of the plasma DHT levels showed significant differences between CM and CF (F_(1,8)_: 31.612; *p* < 0.005), FluM and FluF (F_(1,8)_: 16.216; *p* < 0.01) and FinM and FinF (F_(1,9)_: 10.139; *p* < 0.05), with DHT levels being consistently higher in the male groups (Table 1).

**Fig. 6:**
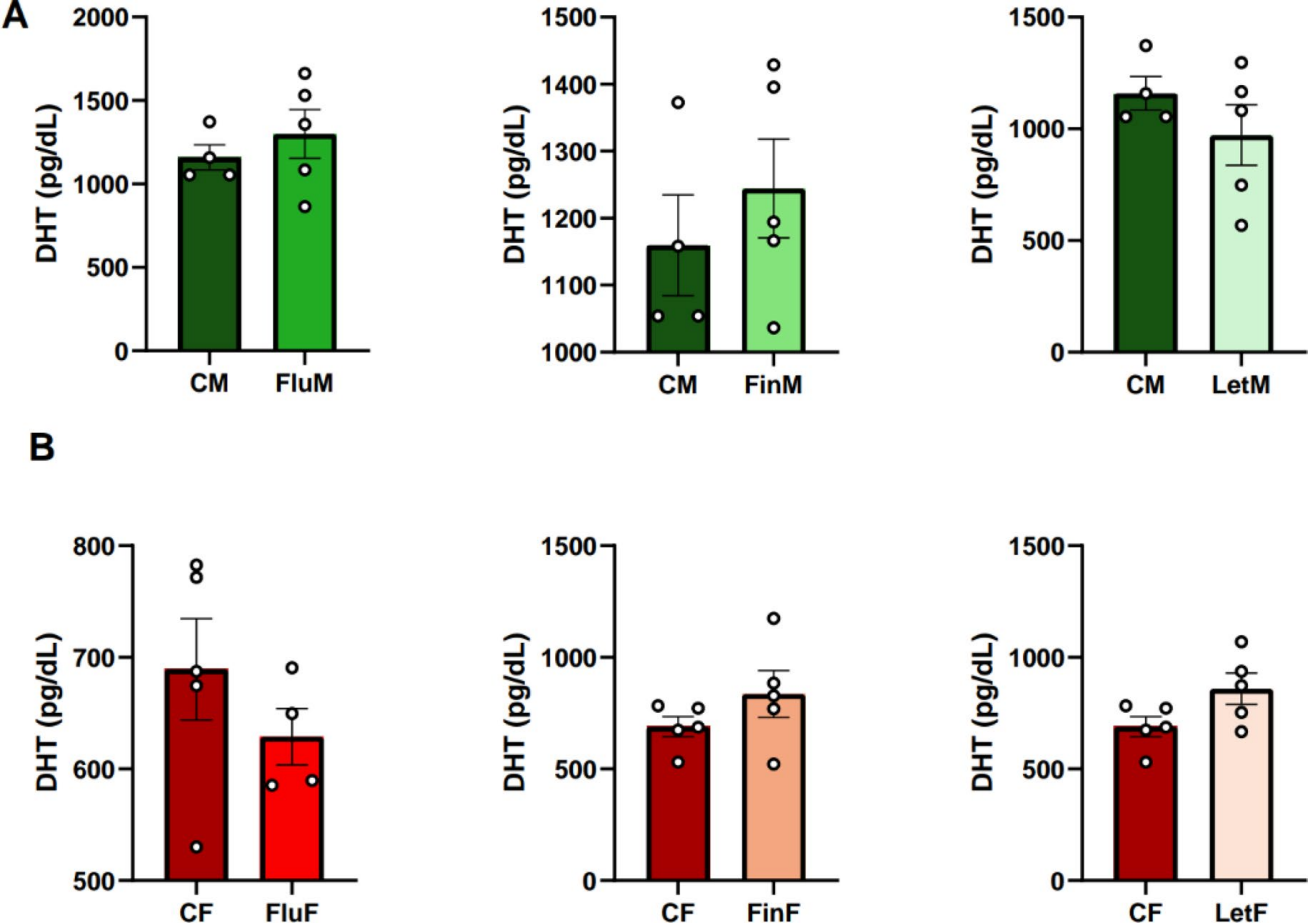
Bar charts of DHT plasma levels, **A**) when male groups, or **B**) female groups were analyzed separately

When each sex was analyzed separately, no significant differences were found (Fig. 6A and B).

#### Estradiol

**Fig. 7:**
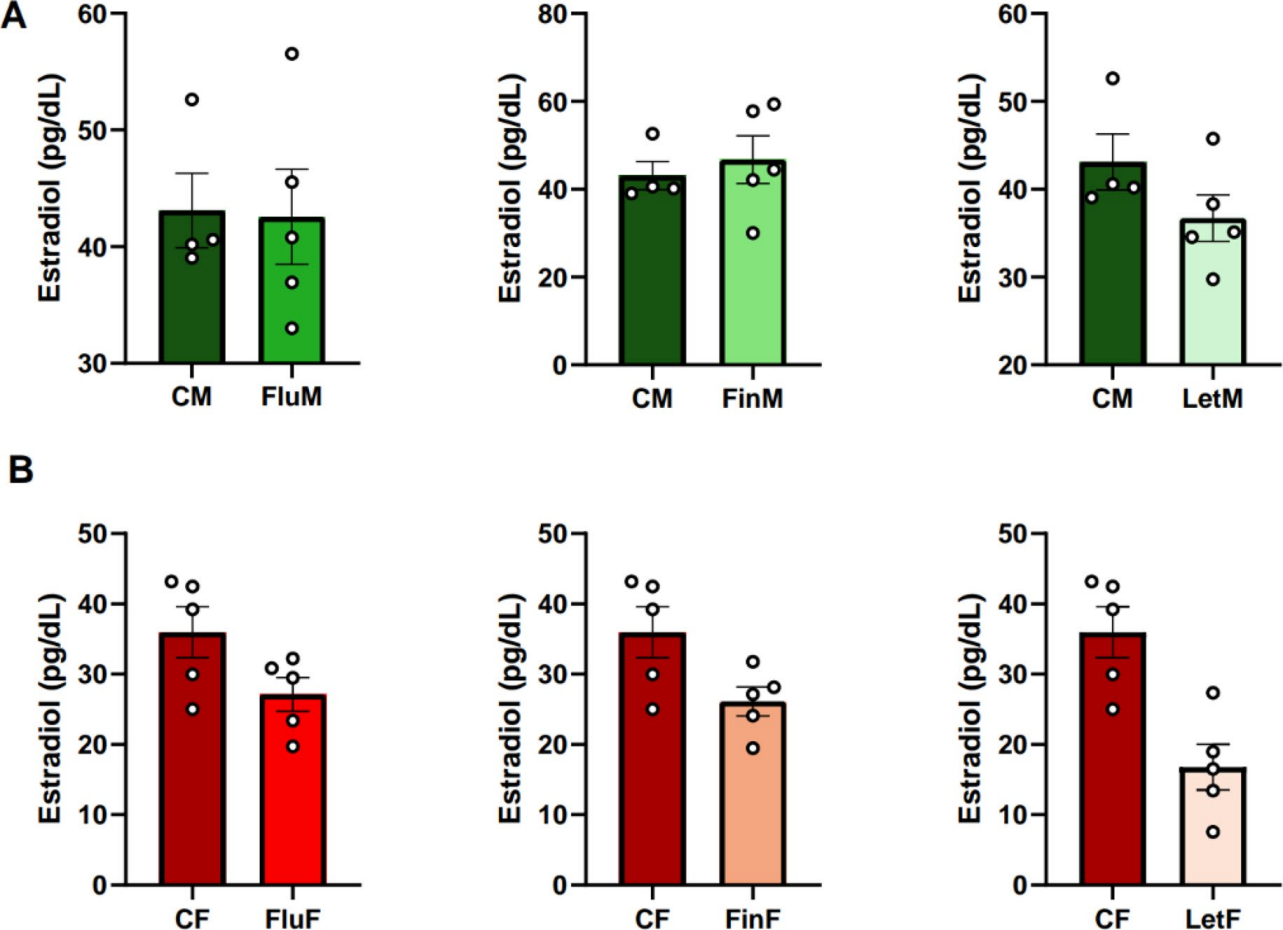
Bar charts of estradiol plasma levels, **A**) when male groups, or **B**) female groups were analyzed separately

Regarding sexual dimorphism, the analysis of the circulating levels of estradiol showed no significant differences between CM and CF, indicating that there is no sexual dimorphism in this parameter. However, significant differences between FluM and FluF (F_(1,9)_: 10.71; *p* < 0.05), FinM and FinF (F_(1,9)_: 12.655; *p* < 0.01), and LetM and LetF (F_(1,9)_: 22.503; *p* < 0.005) were found, with estradiol levels being consistently higher in males compared to females (Table 1). When each sex was analyzed separately, no significant differences were found (Fig. 7A and B).

## Discussion

The most important results of our study revealed that the activity of testosterone and its metabolic pathways during the first five days of life determined the expression of the gonadal steroid receptors in the hypothalamus of adult female rats, while no significant effects were detected in males. Specifically, (1) the inhibition of androgen receptors in female rats produced a significant increase in hypothalamic ERβ, (2) aromatase inhibition up-regulated AR and ERβ in the long term, and (3) the inhibition of 5α-reductase down-regulated ERα. Moreover, our results also showed that the hypothalamic AR, ERα, or ERβ mRNA levels did not differ between male and female rats. In addition to these brain changes, our results also showed that (4) female rats showed a significant decrease in plasma testosterone levels when androgen receptors or their metabolic pathways were inhibited, and (5) of the three treatments, only the inhibition of aromatase provoked a decrease in plasma testosterone levels in males.

Rats with the testicular feminization mutation (Tfm), involving a lack of activity of the AR during the pre and postnatal periods, showed alterations in the morphology and function of various brain structures, including some nuclei in the hypothalamus in males and in females [41–44]. Our results show that when the inhibition of androgenic activity is limited to the first five days of life, it is mainly the female that is affected.

One of the main results showed that inhibition of AR early after birth produced an increase of ERβ in female rats in the long term but not in males. Given that it has been reported that DHT acts through ERβ as well as AR [26, 45] the increase of these receptors in response to the inhibition of ARs may constitute a compensatory mechanism. It could be suggested that in the absence of AR activity, a greater amount of testosterone could be reduced to DHT. Thus, since DHT can act through its metabolite, 3β-diol, on ERβ receptors [26], in the absence of AR activity an upregulation of ERβ expression, due to the increased DHT activity, could/might occur. Although there is not much information on the specific participation of DHT in the development of different brain parameters in females, it has previously been reported that this hormone participates in the programming of sexual differentiation of the hypothalamic melanocortin system that regulates food intake in female mice [6]. Our results show that AR activity is necessary during the first days of life for adequate ERβ expression in female rats in adulthood.

In relation to the effects of 5α-reductase inhibition, it is important to note that the 5α-reductase enzyme has two isoforms, type 1 and type 2 and, of the two, the latter seems to depend on the activity of androgens and is expressed in the brain in the last stage of gestation and during the first days of life, with females presenting higher levels than those of males on postnatal day 12 [46–48]. These characteristics suggest that 5α-reductase type 2 might be a determining factor in the process of sexual differentiation and the programming of different neural networks [46], and given our results, this could be true specifically in females in the first days of life. Contrary to the initial thought that androgens had no effect on the organization and programming of different neural circuits in females during development, in recent decades several authors have confirmed that DHT participates in the organization of structures belonging to the vomeronasal system, which supports reproductive behaviors [5, 49, 50], and in the hypothalamic system that regulates feeding [6, 51]. The interaction between DHT and ERα has been demonstrated by other authors in both the Arc and VMH in female rats [27] and our results demonstrate that the expression of ERα in the hypothalamus in adult female rats depends on 5α-reductase activity early after birth.

Regarding the results of aromatase inhibition, males show higher levels of aromatase than females during development, probably due to the relevant function that aromatase has during the first days of life in determining the differentiation of brain structures involved in reproductive behaviors in males [4]. The aromatase enzyme is present in both males and females during the perinatal period and then gradually decreases [52]. On the other hand, androgen and estrogen receptors are involved in the aromatization of testosterone and participate in the programming of specific neural networks during development [53, 54]. In the present study, we have shown that the inhibition of aromatase activity during the first five days of life produced an increase in hypothalamic AR and ERβ mRNA levels, but only in females. Although the aromatase enzyme is present in both males and females during the perinatal period and then gradually decreases [52], the fact that aromatase inhibition only influenced females is consistent with the results of other authors showing that treatment with estradiol or DHT can increase the expression of aromatase in cultures of XX embryos but not in XY ones [22].

Together, these results as regards the effects of inhibiting the AR and testosterone metabolic pathways show that during the first five days of life, the androgenic activation of the AR and the androgenic metabolic pathways that metabolize testosterone to estradiol or DHT are important for females since a decrease in this activation can program the long-term mRNA levels of AR, ERα and ERβ receptors, possibly through mechanisms that can, in some way, compensate for the functions inhibited.

Regarding the possible existence of sex differences in the hypothalamic mRNA levels of gonadal steroid receptors, our results do not show differences between any group, although the changes experienced in females caused the appearance of differences between males and females in some of the gonadal steroid receptors depending on the treatment. The existence of sexual dimorphism in the expression of these receptors has been reported in some studies [55–59], although this has not been the case in others [60]. The inconsistency of the reported data regarding sex differences in the expression of gonadal steroid receptors in the hypothalamus is probably due to the different methodological approaches used or differences between the species studied.

Finally, the data previously obtained on plasma testosterone levels could complement our findings obtained on hypothalamic gonadal steroid mRNA levels in females. The fact that testosterone levels in adult females decreased in all treatments given in this study indicates, once more, the vulnerability of females to a lack of testosterone activity, either through its receptors or its metabolic pathways, during the first days of life. Since other authors have shown that the expression of estrogen receptors in different hypothalamic nuclei is influenced by the levels of estrogens or androgens [27], we could suggest, in light of our results, that the decrease in testosterone levels, caused by the lack of androgenic and estrogenic activity in the first days of life, could influence the expression of gonadal steroid receptors in the adult stage in females. As regards males, it is difficult to explain why testosterone levels also decrease due to aromatase inhibition. However, this inhibition does not affect the expression of gonadal steroid receptors in the hypothalamus. Future research will be needed to unravel whether plasma levels of gonadal hormones might be part of the mechanism that differentially influences the expression of gonadal hormone receptors in the hypothalamus.

Just as it may have been surprising in the 1970s that the masculinization of the rat brain in certain neural systems was produced by the aromatization of testosterone to estradiol, the results obtained in our study may also be surprising because they demonstrate that the expression of gonadal steroids receptors in the hypothalamus in adulthood depends on the androgen activity during the first five days of life in females, but not in males. Considering our results, it could be suggested that the expression of gonadal steroid receptors in the hypothalamus is vulnerable to the action of testosterone and its metabolic pathways during the first days of life in female rats but not in males. Therefore, our results underline the need for further research to accurately explain the role of gonadal hormones during development in programming hypothalamic circuits in males and females.

## Conclusion

The early stages of development are very vulnerable periods in which it is crucial to maintain the balance of neurohormonal systems to optimize proper brain development. This study has shown that androgen activity during the first five days of life is crucial to ensure the appropriate developmental pattern of the expression of gonadal receptors in the female hypothalamus. The activity of estrogen and androgen receptors in the hypothalamus supports the expression of different behaviors, so any alteration in their programming could result in behavioral alterations. Therefore, it is essential to always consider the action of androgens on the programming of the neural systems that support behaviors such as reproduction or feeding and always in both sexes.

## Data Availability

The datasets generated during and/or analysed during the current study are available from the corresponding author on reasonable request.
